# Epidemiology and drug allergy results in children investigated in allergy unit of a tertiary-care paediatric hospital setting

**DOI:** 10.1186/s13052-019-0753-4

**Published:** 2020-01-10

**Authors:** A. Piccorossi, G. Liccioli, S. Barni, L. Sarti, M. Giovannini, A. Verrotti, E. Novembre, F. Mori

**Affiliations:** 1 Department of Pediatrics, San Salvatore Hospital, University of L’Aquila, L’Aquila, Italy; 2 Allergy Unit, Anna Meyer Children’s Hospital, Florence, Italy

**Keywords:** Pediatrics, Drug hypersensitivity reaction, Allergy tests, Epidemiology

## Abstract

**Background and objective:**

Drug Hypersensitivity Reactions (DHRs) are considered adverse effects of medications that resemble allergy symptoms. The reported positive clinical history of pediatric drug reactions is about 10%, however, after allergy investigations, only a small percent is confirmed as hypersensitivity.

The aim of this study was to analyze the clinical history, allergy work-up results and sensitization profile of children and adolescents referred to our Allergy Unit for suspected DHRs.

**Methods:**

The study evaluated data related to a group of children with a positive history of drug reactions during a two-year period. The allergy work-up consisted of in vivo and in vitro tests, in accordance with the recommendations of the ENDA/EAACI guidelines.

**Results:**

Data from a group of 637 patients [348 M (54.6%); 289 F (45.4%)] were retrospectively analyzed. Beta lactams (BLs) were the most common drugs involved in the reported clinical history, followed by non-steroidal anti-inflammatory drugs (NSAIDs). Severe cutaneous adverse reactions (SCARs) were most frequently observed during BL treatment. The confirmation of BL hypersensitivity was higher for immediate reactions (IRs) [9.4%; 5.1% through positive skin tests (STs) and 5.5% through drug provocation test (DPT)] compared to non-immediate reactions (non-IRs) (8.1%; 2.2% through STs and 6.2% through DPT). A higher number of positive results was obtained for BLs and macrolides when the tests were performed within 12 months after the index reaction (*p* < 0.05). During DPTs with amoxicillin-clavulanic acid, four hypersensitivity reactions (including one anaphylaxis) occurred despite negative STs.

**Conclusion:**

Our data demonstrated that only 9.1% of patients resulted in being positive to allergy tests which is in line with the data in literature. An allergy work-up is mandatory for excluding suspected hypersensitivity.

## Background

Evaluation of Drug Hypersensitivity Reactions (DHRs) is a common topic of debate at a pediatric age. DHRs are defined as adverse effects of medications that clinically resemble allergies [1].

Data in literature reports about a 10% prevalence of a positive history of pediatric drug reactions, but only a small percent is confirmed after an allergy work-up [2, 3].

Furthermore, drug reactions, especially during childhood, are non-immediate and often require differential diagnosis with viral infections that could mimic skin eruptions resembling that of DHRs, and at the same time cause a drug interaction [4]. Little data are available on the real incidence and confirmatory investigations of DHRs in pediatric patients. A reason is due to the facts that real sensitivity and specificity of in vivo and in vitro tests are difficult to determine, especially in the pediatric population, where they are not standardized for each class of drugs [3]. Therefore, a drug provocation test (DPT) is mandatory to confirm or exclude drug hypersensitivity. Moreover, a correct diagnostic workup represents the only way to offer a safe alternative drug in case of true drug allergies and “de-label” false drug allergies in children, that are associated with inappropriate alternative treatments.

Prescribed drugs differ between children and adults, moreover for many kinds of drugs there are no therapeutic alternatives in the pediatric population and for special categories of patients some drugs are not equally as effective (e.g. several categories of antibiotics in cystic fibrosis) [5]. The most frequent classes of drugs reported as responsible of hypersensitivity reactions are beta lactams (BLs) and non-steroidal anti-inflammatory drugs (NSAIDs) [3]. The aim of our study was to evaluate the clinical history, diagnostic work-up results and sensitization profile of children referred to our Allergy Unit for suspected DHRs over a two-year period.

## Methods

Data were retrospectively collected from patients with a positive history of drug reaction referred to the Allergy Unit of Anna Meyer Children’s Hospital (Florence, Italy) over a two-year period (1st January 2017 – 31st December 2018). According to the hospital ethic committee form, all children’s parents signed an informed consent to the processing of clinical data for future research studies. Patients who did not give the consent were excluded from the present study.

Patients with a history of drug reactions underwent an allergy work-up consisting of skin tests (STs) (skin prick test-SPT; intradermal test-IDT; patch tests-PT), and in vitro tests for the quantification of specific IgE (sIgE) antibodies. The evaluation was performed in accordance with the current recommendations of the ENDA/EAACI guidelines for antibiotics, NSAIDs and local anesthetics [3].

SPTs were conducted using a dilution of antibiotic powder for intravenous solution or undiluted in case of local anesthetics (see Table 1) prepared immediately before testing; a reaction was considered positive when a wheal ≥ 3 mm surrounded by erythema was observed 15 min after the administration of 1 drop of reagent on the volar surface of the forearm skin. In case of negative SPTs, IDTs were performed, by injecting 0.03 ml of solution into the volar surface of the forearm; an increase in wheal diameter greater than 3 mm with infiltration surrounded by erythema was considered positive. Readings were obtained after 20 min and at 24, 48, 72 h after the injection. Histamine (ALK-Abellò, Milan, Italy: 1 mg/mL) was used as positive control. Normal saline was used as negative control. PTs were freshly prepared at the recommended concentrations (see Table 1) in petrolatum, with readings 15 min and 24–48-72 h after removal of the strips applied on the children’s backs for 48 h. Petrolatum was used as a negative control. A PT was defined as positive when an infiltrate was detected. In vivo tests (ID and SPT) were made at least 6 weeks after recovery and 4 weeks after stopping corticosteroid (CCS) treatment, especially in case of Drug Rash with Eosinophilia and Systemic Symptoms (DRESS) or Stevens Johnson Syndrome/Toxic Epidermal Necrolysis (SJS/TEN), where children treated with CCS needed to be out of therapy for at least 4 weeks.

**Table 1:** Standardized concentrations for STs and DPTs. IU: International Units; IM: intramuscular; PO: orally administered; IV: intravenous; SPT skin prick test; ID intradermal test; PT patch test; DPT drug provocation test

Drugs	Skin reaction		SPT	ID	PT	DPT
	IR	non-IR				
BETALACTAMS	Urticaria 42.2% Erythematous Rash 18.7% Maculopapular rash 8.6% Measles like rash 8.6% Not defined rash 7.8% Urticaria+ Angioedema 3.9% Angioedema 3.9% Micropapular rash 3.9% Itching 1.6% Exfoliative Dermatitis 0.8%	Urticaria 36.2% Erythematous rash 20.4% Maculopapular rash 14.4% Micropapular rash 8.1% Not defined rash 6.8% Angioedema 6.3% Urticaria+ Angioedema 5% Itching 1% Exfoliative Dermatitis 0.9% Measles like rash 0.9%	10.000 IU	10.000 IU	5%	IM 600.000 IU < 27 kg IM 1.200.000 IU > 27 kg
Benzylpenicillin						
Amoxicillin			20 mg/mL	20 mg/ml	5%	PO: 25 mg/kg/dose
Amoxicillin-Clavulanic Acid			20 mg/mL	20 mg/ml	5%	PO: 25 mg/kg/dose
Cephalosporins			2 mg/mL	2 mg/ml	5%	Ceftriaxone IV: 80 mg/kg/dose Cefpodoxime PO: 4 mg/kg/dose
MACROLIDES	Urticaria 35% Erythematous rash 30% Angioedema 10% Not defined rash 10% Urticaria+ Angioedema 5% Micropapular rash 5%	Urticaria 47% Erythematous rash 23% Micropapular rash 15% Maculopapular rash 6% Angioedema 3% Urticaria+ Angioedema 3% Itching 3%	50 mg/mL	0.5–0.05 mg/mL		PO: 7.5 mg/kg/dose
Clarithromycin						
Azithromycin			100 mg/mL	0.1–0.01 mg/mL		PO: 10 mg/kg/dose
Trimethoprim-Sulphametoxazole			80 mg/mL	0.8 mg/mL	5%	PO: 3 mg/kg/dose
Ciprofloxacin			up to 2 mg/mL	up to 0.006 mg/mL	10–25%	IV: 6 mg/kg/dose
Local anesthetics	Angioedema 65% Itching 15% Urticaria 10% Urticaria+ Angioedema 5% Localized Erythematous Rash 5%	Angioedema 53.8% Erythematous Rash 23.1% Maculopapular rash 7.7% Fixed Drug Eruption 7.7% Erythematous Rash+ Angioedema 7.7%	Undiluted	1/10 diluted	–	1) 0.1 mL undiluted 2) 1 mL undiluted 3) 2 mL undiluted
NSAIDs	Angioedema 32.9% Erythematous Rash 18.2% Urticaria 17.1% Itching 14.6% Urticaria+ Angioedema 13.4% Not defined rash 2.7% Maculopapular rash 1.1%	Angioedema 45.5% Urticaria 17.4% Urticaria+ Angioedema 14.5% Micropapular rash 5.4% Erythematous rash 5.4% Vesicular rash 5.4% Not defined rash 2.7% Petechiae 2.7%	–	–	–	Paracetamol PO:15 mg/kg/dose Ibuprofen PO: 10 mg/kg/dose ASA PO: 15–20 mg/kg/dose Etoricoxib PO: 60 mg/dose

The standardized concentrations of drugs used for skin testing are summarized in Table 1.

When a reaction occurred within 1 h after the last drug administration it was classified as an immediate reaction (IR), i.e. from urticaria to anaphylaxis, while in case of a reaction appearing more than 1 hour later, it was considered a non-immediate (non-IR), i.e. from maculopapular exanthemas (MPEs) to severe cutaneous adverse reactions (SCARs). In case of reactions within 6 h after the last drug intake and negativity of the previous tests, a DPT was conducted with the culprit.

All DPTs were open-challenge and performed after obtaining signed parental informed consent. In case of anaphylaxis or delayed severe reactions such as DRESS or SJS/TEN, DPTs with the culprit drug were not carried out, and in selected cases a DPT with an alternative drug was performed.

In the group with a history of reactions to NSAIDs, patients who were cross-intolerant due to a positive DPT with acetylsalicylic acid (ASA), were admitted to a challenge with a cycloxigenase-2 (COX2) selective drug when over the age of 12.

BLs sIgEs for the hapten c1 (penicilloyl G), c2 (penicilloyl V), c5 (ampicilloyl), c6 (amoxicilloyl), c7 (cefaclor) were obtained using fluoroimmunoassay (ImmunoCAP Thermo-Fisher, Uppsala, Sweden). Results were considered “positive” with values ≥ 0.35 kUA/L and “borderline” between 0.10 and 0.35 kUA/L.

For clarithromycin, azithromycin, cephalosporins, trimethoprim-sulphamethoxazole, vancomycin and quinolones, the sIgEs were determined using radioimmunoassay (Sepharose 6B epoxy- activate as solid phase). The radioactivity ratio, obtained from antibodies bound in the solid phase to the total amount of radioactive reactant introduced, was considered positive in case of values at least 3 times higher than the normal serum level.

In the case of NSAIDs, in vivo and in vitro tests are not standardized, thus an oral DPT was performed directly.

Before starting the DPT, patients were checked to exclude possible underlying infections and/or pre-existing eruptions with skin involvement (i.e. acute urticaria).

The dosing regimens for the DPTs were calculated based on the patient’s weight (Table 1). Patients had to be off antihistaminic therapy for at least 7 days and corticosteroid or immunosuppressive therapy for at least 30 days.

The DPTs for antibiotics consisted of a two-day test; on the first day, a cumulative dose was split into 3 doses (1/10, 2/10, 7/10) and administered at 30-min intervals (by oral route except for intravenous antibiotics and subcutaneous local anesthetics); on the following day the complete dose was given in one administration in case of oral antibiotics. After the last administration, patients were kept under observation to monitor for adverse events at least every 2 and 3 h on the first and second day respectively.

DPTs for NSAIDs were performed with incremental dose administration (1/10–2/10–7/10) every 30 min followed by a three-hour observation.

When any objective symptoms occurred (i.e. cutaneous and/or respiratory symptoms or alterations in vital signs like rhythm alterations, reduced oxygen saturation or hypotension), the procedure was discontinued and the symptoms promptly evaluated and treated.

In patients with a history of non-immediate reactions (non-IRs) to antibiotics, oral administration of the culprit was prolonged for a further 5 days at home [6], and the families supplied with antihistamines and oral corticosteroids. In case of adverse events, parents were advised to stop treatment and to communicate the reaction by sending a photo by email in case of skin eruptions. DPTs for local anesthetics included subcutaneous injection of the undiluted drug into the extensor arm region at the width of a hand above the elbow at 30-min intervals.

### Statistical analysis

Data analysis was performed using IBM SPSS version 22. Quantitative variables were summarized as mean and standard deviations (SDs); categorical variables were indicated as a number (%). Distributions of qualitative variables between subgroups were compared using the chi-square test. Statistical significance was considered for all tests as *p* < 0.05.

## Results

Data from a group of 637 patients [348 M (54.6%); 289 F (45.4%)] were retrospectively analyzed.

The mean age was 10.1 years (range 1–18 years). The patients’ features are summarized in Table 2.

**Table 2:** Demographic characteristics of patients

Suspected drug	No. of patients	Age	Sex	Atopy	Time between reaction and tests (months)
Beta Lactams	386	7.3 ± 11.4	M 216 F 170	114	24.3 ± 33.8
Macrolides	61	10.4 ± 4.2	M 28 F 33	17	22 ± 53.3
Glycopeptides	4	8.2 ± 11.5	M 2 F 2	2	4.5 ± 5.8
Trimethoprim-sulfamethoxazole	6	11.5 ± 15.4	M 4 F 2	3	35.6 ± 83.3
Quinolones	3	14.3 ± 1.5	M 2 F 1	1	6.7 ± 11.3
NSAIDs	143	9.2 ± 13.8	M 75 F 68	84	27.1 ± 60.5
Local Anesthetics	34	9.2 ± 12.5	M 21 F13	23	9.9 ± 19.7

**Fig. 1:**
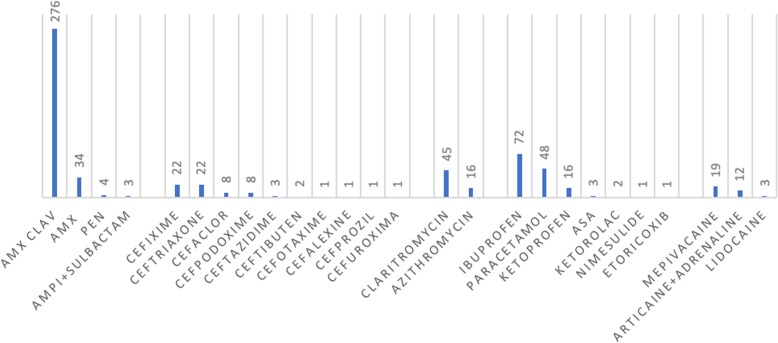
Drugs involved in a history of referred reactions (absolute numbers)

Among the BLs, amoxicillin-clavulanic acid was most frequently reported (Fig. 1).

**Fig. 2:**
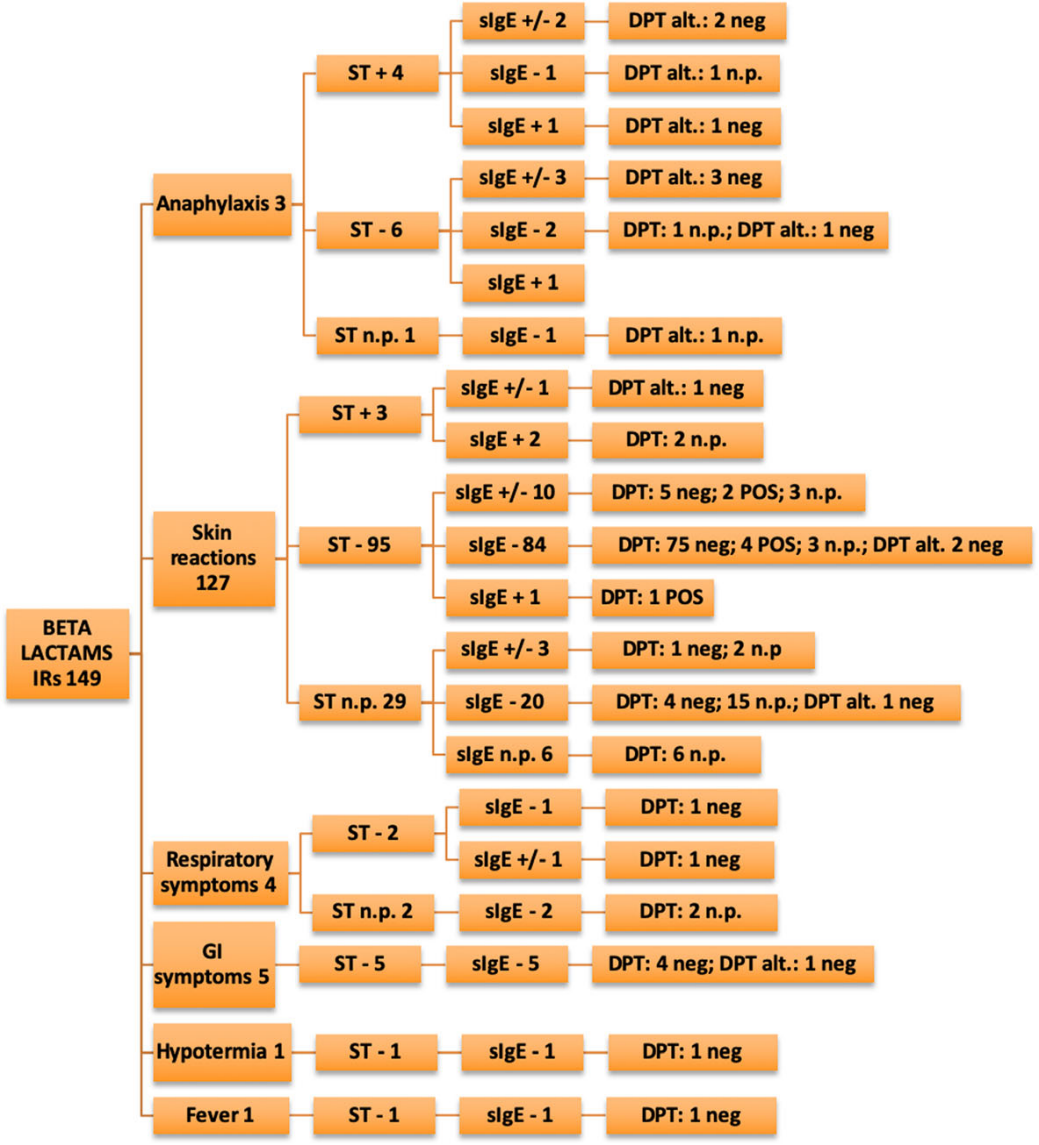
Allergy work-up results for the main drug classes: betalactams immediate-reactions IRs: immediate reactions; Non-IRs: non-immediate reactions; STs: Skin tests; DPTs: drug provocation tests; Alt: alternative drug; NSAIDs: Non-steroidal anti-inflammatory drugs; ASA: acetylsalicylic acid; COX: cyclooxygenase; U/A: urticaria/angioedema; SCAR: Severe Cutaneous Adverse Reactions; DRESS: Drug Reaction with Eosinophilia and Systemic Symptoms; SSLR: Serum Sickness–Like Reaction; SJS: Stevens-Johnson syndrome; THR: thrombocytopenia; GI: gastrointestinal; neg: negative; POS: positive; n.p.: not performed

**Fig. 3:**
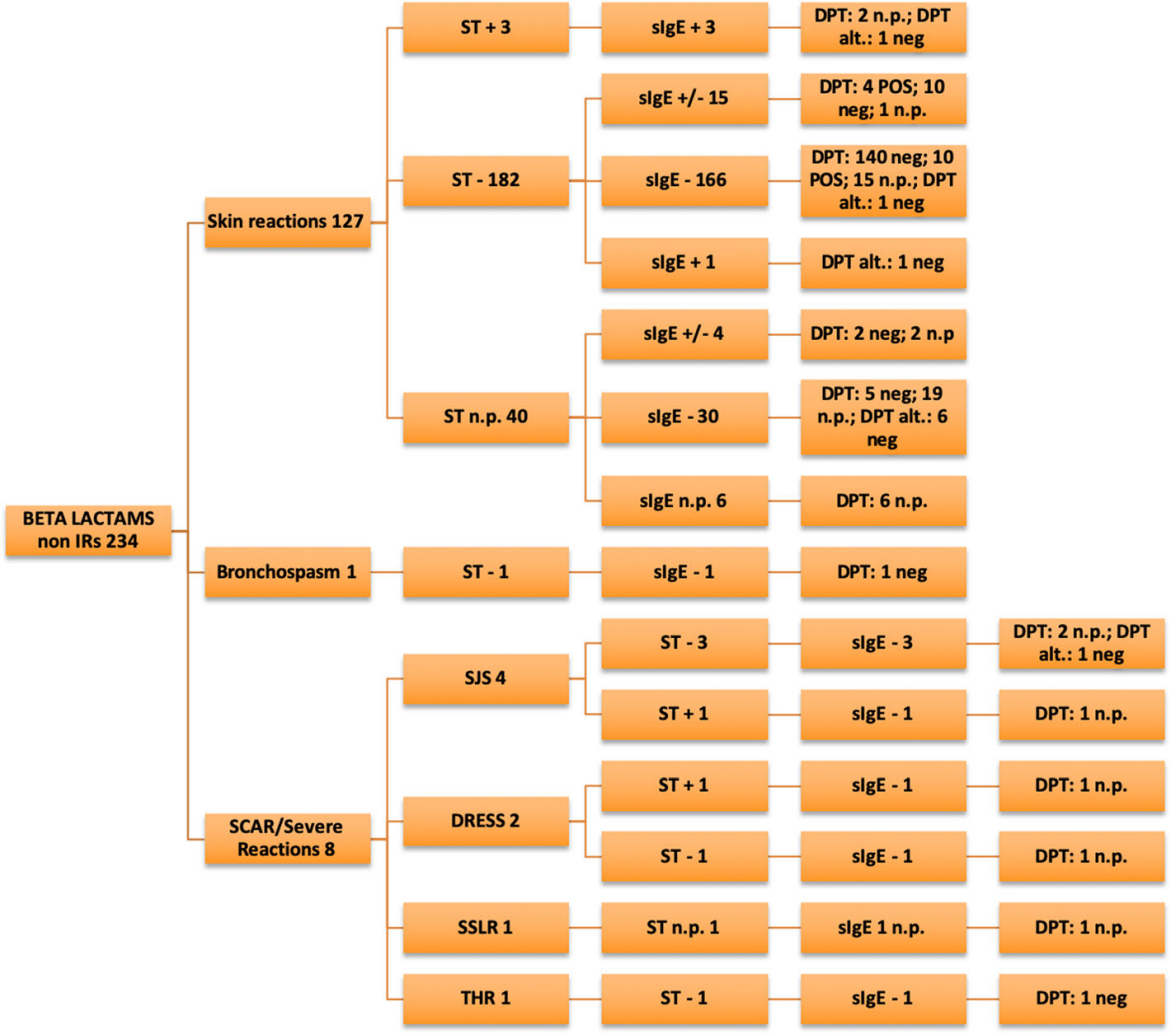
Betalactams non-immediate reactions

**Fig. 4:**
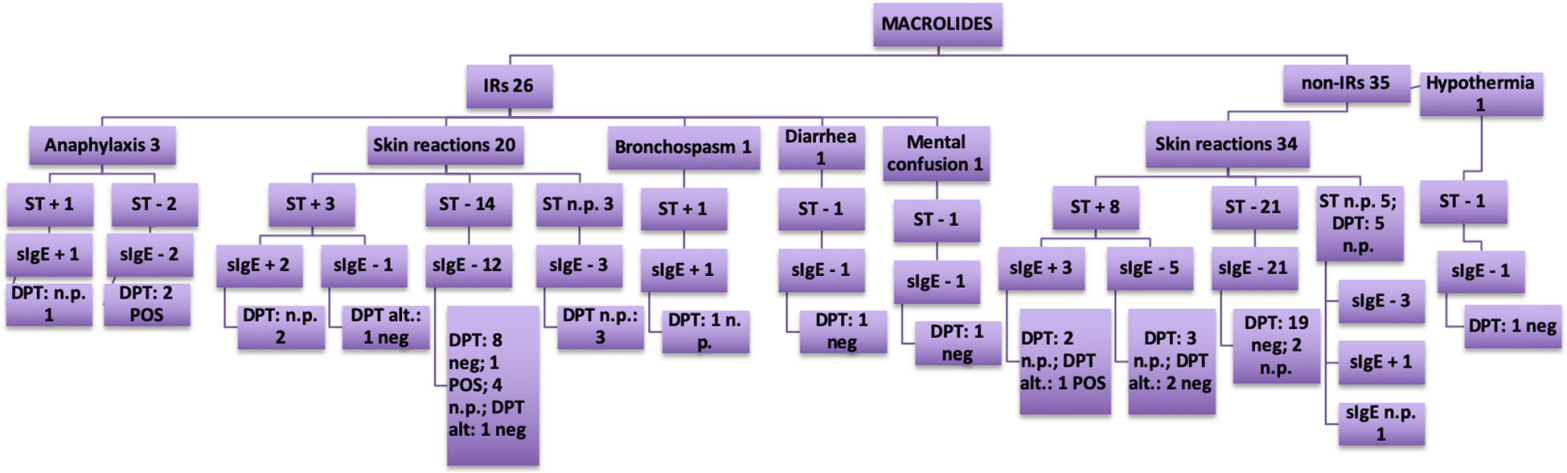
Macrolides

**Fig. 5:**
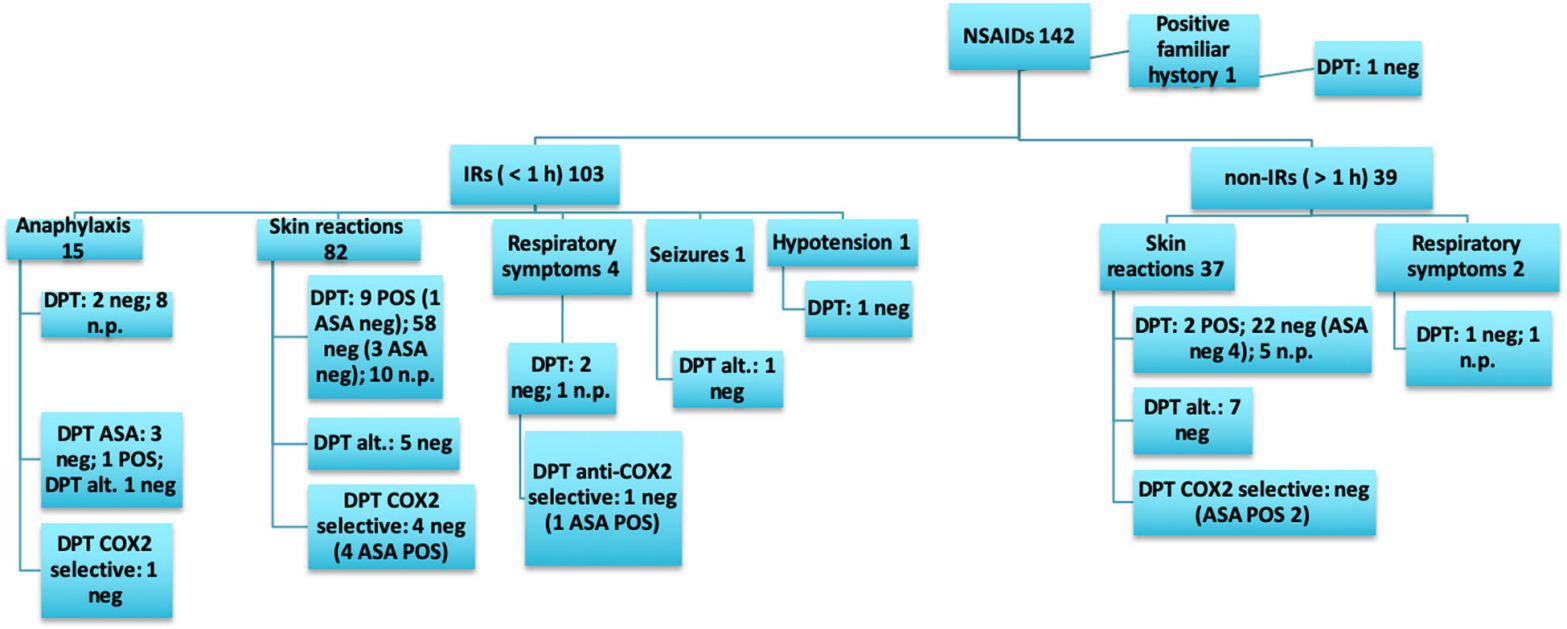
Non-steroidal anti-inflammatory drugs

**Fig. 6:**
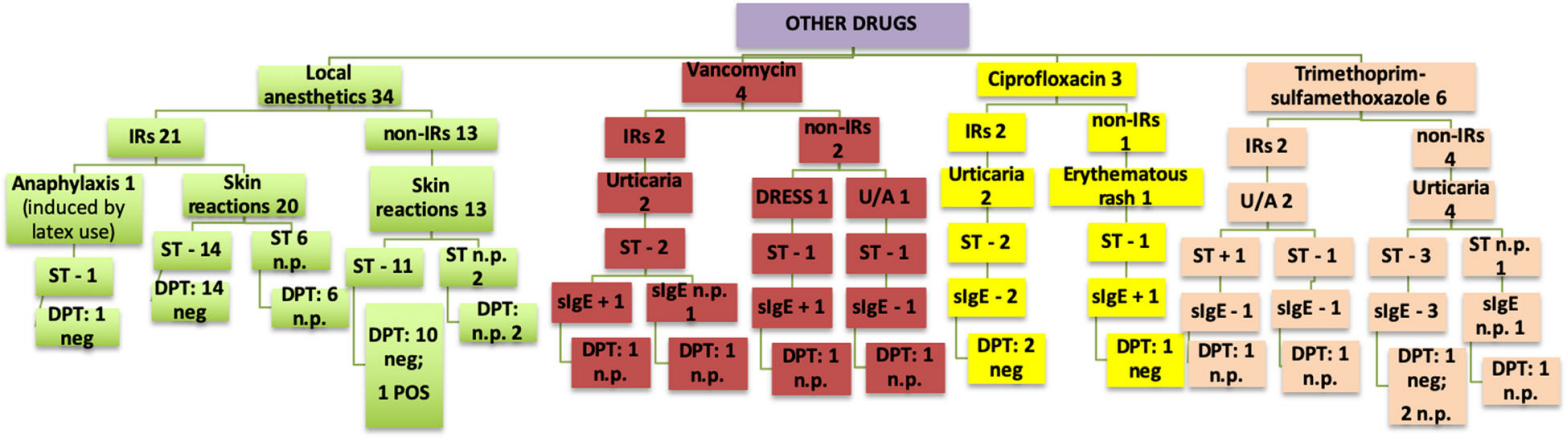
Other drugs

The data analysis showed a slight male predominance in the examined group (54.6% vs. 45.4%). The test results are described in Tables 3, 4, 5, 6) and illustrated in Figs. 2, 3, 4, 5 and 6.

**Table 3:** Summary of results for IRs: Antibiotics and Local Anesthetics

	*ST+*	*ST-*	*DPT+*	*DPT-*	*DPT Alt.Neg*
BLs (no. 149)					
Anaphylaxis (no. 11)	4/11 (36.4%)	6/11 (54.5%)	–	–	7/11 (63.6%)
Skin Reactions (no. 127)	3/127 (2.4%)	95/127 (74.8%)	7/127 (5.5%)	85/127 (66.9%)	5/127 (4%)
Respiratory Symptoms (no. 4)					
-Cough (no. 3)	–	2/4 (50%)	–	2/4 (50%)	
-Bronchospasm (no. 1)					
Gastrointestinal Symptoms (no. 5)					
-Vomiting (no. 2)	–	5/5 (100%)	–	4/5 (80%)	1/5 (20%)
-Diarrhea (no. 3)					
Hypothermia (no. 1)	–	1/1 (100%)	–	1/1 (100%)	–
Fever (no. 1)	–	1/1 (100%)	–	1/1 (100%)	–
Positive Familiar History (no. 3)	–	3/3 (100%)	–	3/3 (100%)	–
Macrolides (no. 26)					
Anaphylaxis (no. 3)	1/3 (33.3%)	2/3 (66.7%)	2/3 (66.7%)	–	–
Skin Reactions (no. 20)	3/20 (15%)	14/20 (70%)	1/20 (5%)	9/20 (45%)	2/20 (10%)
Respiratory Symptoms (no. 1)					
-Bronchospasm (no. 1)	1/1 (100%)	–	–	–	–
Mental Confusion (no. 1)	–	1/1 (100%)	–	1/1 (100%)	–
Glycopeptides (no. 2)					
Skin reactions (no. 2)	*–*	2/2 (100%)	–	*–*	*–*
Trimethoprim Sulfamethoxazole (n °2)					
Skin reactions (no. 2)	1/2 (50%)	1/2 (50%)	–	–	–
Fluoroquinolones (no. 2)					
Skin reactions (no. 2)	–	2/2 (100%)	–	2/2 (100%)	–
Local Anesthetics (no. 21)					
Anaphylaxis (no. 1)	1/1 latex	1/1 (100%)	–	1/1 (100%)	*–*
Skin Reactions (no. 20)	*–*	14/20 (70%)	–	14/20 (70%)

**Table 4:** Summary of results for IRs: NSAIDs

NSAIDs (no. 103)	*DPT+*	*DPT-*	*DPT Alt. Neg*	*DPT ASA-*	*DPT ASA+*	*DPT COX2 selective -*	*DPT n.p.*
Anaphylaxis (no. 15)	–	2/15 (13.3%)	1/15 (6.7%)	3/15 (20%)	1/15 (6.7%)	1/15 (6.7%)	8/15 (53.3%)
Skin Reactions (no. 82)	9/82 (11%)	58/82 (70.7%)	5/82 (6.1%)	4/82 (4.9%)	4/82 (4.9%)	4/82 (4.9%)	10/82 (12.2%)
Respiratory Symptoms (no. 4)							
-Cough (no. 2)	–	2/4 (50%)	–	–	1/4 (25%)	1/4 (25%)	1/4 (25%)
-Bronchospasm (no. 2)							
Seizures (no. 1)	–	1/1 (100%)	–	–	–	–	–
Hypotension (no. 1)	–	1/1 (100%)	–	–	–	–	–

**Table 5:** Summary of results for non-IRs: Antibiotics and Local Anesthetics

	*ST+*	*ST-*	*DPT+*	*DPT-*	*DPT Alt. Neg*	*DPT Alt. Pos*
BLs (no. 234)						
Skin Reactions (no. 225)	3/225 (1.3%)	182/225 (80.9%)	14/225 (6.2%)	157/225 (69.8%)	9/225 (4%)	–
Respiratory Symptoms (no. 1)						
-Bronchospasm (no. 1)	–	1/1 (100%)	–	1/1 (100%)	–	–
SJS (no. 4)	1/4 (25%)	3/4 (75%)	–	–	–	–
DRESS (no. 2)	1/2 (50%)	1/2 (50%)	–	–	–	–
SSLR (no. 1)	–	–	–	–	–	–
Thrombocytopenia (no. 1)	–	1/1 (100%)	–	1/1 (100%)	–	–
Macrolides (no. 35)						
Skin Reactions (no. 34)	8/34 (23.5%)	21/34 (61.7%)	–	19/34 (55.9%)	2/34 (5.9%)	1/34 (2.9%)
Hypothermia (no. 1)	–	1/1 (100%)	–	1/1 (100%)	–	–
Glycopeptides (no. 2)						
Skin reactions (no. 2)	*–*	2/2 (100%)	–	*–*	*–*	*–*
Trimethoprim Sulfamethoxazole (n °4)						
Skin reactions (no. 4)	–	3/4 (75%)	–	1/4 (25%)	–	–
Fluoroquinolones (no. 1)						
Skin reactions (no. 1)	–	1/1 (100%)	–	1/1 (100%)	–	–
Local Anesthetics (no. 13)						
Skin Reactions (no. 13)	*–*	11/13 (84.6%)	1/13 (7.7%)	10/13 (76.9%)	*–*	–

**Table 6:** Summary of results for non-IRs: NSAIDs

NSAIDs (no. 39)	*DPT+*	*DPT-*	*DPT Alt. Neg*	*DPT ASA-*	*DPT ASA+*	*DPT COX2 selective -*	*DPT n.p.*
Skin Reactions (no. 37)	2/37 (5.4%)	22/37 (59.4%)	7/37 (18.9%)	4/37 (10.8%)	2/37 (5.4%)	2/37 (5.4%)	5/37 (13.5)
Respiratory Symptoms (no. 2)							
-Dyspnea (no. 2)	–	1/2 (50%)	–	–	–		1/2 (50%)
Positive Familiar History (no. 1)	–	1/1 (100%)	–	–	–	–	–

*DPT alt.* drug provocation test with an alternative drug

*N.p.* not performed

In our study, BLs were the drugs most commonly involved in the reported reactions followed by NSAIDs.

With the BL reactions, cutaneous symptoms occurred with greater frequency, mostly urticarial rashes. Severe non-IRs were observed most frequently during the course of BL treatments, with Steven Johnson Syndrome (SJS) occurring in 4 cases (3: amoxicillin-clavulanic acid, 1: ceftriaxone).

In the BL group, STs were positive in 3.2% of patients (12/386), as per the following distribution by pool of symptoms: anaphylaxis 36.4% (4/11), skin involvement 1.7% [6/352; (IRs:3/127–2.3%; non-IRs:3/225–1.3%)], severe reactions 25% (2/8). All patients with positive SPTs to amoxicillin-clavulanic acid were also positive to amoxicillin alone, so we excluded hypersensitivity to clavulanic acid. We obtained a positive PT in one patient with a history of SJS and a positive IDT reading at 72 h; in one case of DRESS, a positive IDT reading at 72 h was observed. The diagnosis of BL hypersensitivity was confirmed with DPTs with the culprit drug in 5.4% (21/386) of patients. On analyzing the IRs, hypersensitivity was confirmed in 9.4% (14/149) of patients, and with non-IRs we had positive results in 8.1% (19/234) of cases. We also compared results of DPT and STs. Excluding anaphylaxes and SCARs, we found that, in IRs patient’s group, there were 7 patients with false negative STs results (negative predictive value 92%) and in non-IRs 14 false negative STs results (negative predictive value 92%).

In the macrolide group, 73.4% of patients had a history of reactions to clarithromycin. The STs were positive in 19.7% (12/61) of patients. The DPTs were positive in 3/61 cases (4.9%); two of these patients reported a suspected history of mild anaphylaxis to clarithromycin, the first had a history of several cutaneous reactions and on one occasion dyspnea, the second had urticaria with cough. In both cases, due to negative STs and sIgE results with not a particularly convincing history of reactions, DPTs were performed. In the non-IR group 19/19 (100%) the DPTs were negative. Overall, in the macrolide group, considering positivity of both STs and DPTs, we had evidence of hypersensitivity in the 30.7% (8/26) of patients among the IRs group, and in 22.8% (8/35) among the non-IRs group.

Patients with a history of IRs to BLs and macrolides were divided into two categories based on latency between reaction and allergy investigations (before or after 12 months: < 12 m or > 12 m). Diagnostic confirmation of hypersensitivity was compared in both groups. In particular, we obtained a higher rate of positive results when investigations before 12 months were carried out (BLs < 12 m: 40% vs. > 12 m: 4.3%; macrolides < 12 m: 33% vs. > 12 m: 16.7%). Tests performed within 12 months were statistically significant for both categories (*p* < 0.05).

One case of DRESS induced by vancomycin was reported; all the STs resulted negative, including PTs at 20% in petrolatum.

In one patient with a history of cutaneous IR to trimethoprim-sulfamethoxazole, the IDT reading at 20 min was positive.

NSAID reactions most frequently involved ibuprofen (Fig. 1), and a positive DPT with the culprit drug was obtained in 11.4% (11/96) of patients. This category reported the highest percentage of anaphylaxis (2.3%), the drugs involved were the following: ibuprofen (46.6%, 7/15), paracetamol (26.7%, 4/15), ketoprofen (20%, 3/15) and ketorolac (6.7%, 1/15). Only 19 out of 117 DPTs were performed with ASA and 10 out of 19 were positive (52.6%). In the ASA tested group the mean age was 11.1 years and 14 out of 19 patients (73.7%) had a clinical history of atopy (11 cases inhalant allergy, 1 food allergy, 1 atopic dermatitis, and 1 venom allergy); subjects with positive results were all adolescents and three had a previous diagnosis of rhino-conjunctivitis and one of asthma.

As regards local anesthetics, patients had 65% of negative ST results and the DPT was only positive in one case (2.7%). In one patient with history of anaphylaxis during dental procedures, a positive ST with latex extract was obtained, but the ST with mepivacaine (the local anesthetic used) was negative. Consequently, a DPT with mepivacaine was possible, ruling out the hypothesis of drug involvement.

Table 1 contains the percentages and types of skin reactions: urticaria was more frequently associated with a reaction to antibiotics, whereas angioedema was more closely linked to NSAIDs and local anesthetics.

Table 7 contains the types of reactions occurring during the DPTs in relation to the time latency and doses.

**Table 7:** Reactions occurring during DPTs with corresponding times and dose reactions

Drug Categories	IRs	NO.	TI (m)	Dose	Non- IRs	NO.	TI (h-d)	Dose
BLs (20)	Ana	1 (5%)	90	7/10	MPE	5 (25%)	4:6–8 h/ 2d	100%
	U	4 (20%)	< 60	7/10			1: 6 h/ 3d	
	ER	1 (5%)	< 60	7/10	U	4 (20%)	2:6-8 h/4d 1: 6–8 h/1d 1^a^	1:7/10 3:100%
					ER	3 (15%)	6–12 h/2d	100%
					ER+ Diarrhea	1 (5%)	6 h/3d	100%
					ED	1 (5%)	8–10 h/1d	7/10
Macrolides (3)	U	2 (66.7%)	< 60	1/10 7/10	–	–	–	–
	ER + Diarrhea	1 (33.3%)	30	7/10	–	–	–	–
NSAIDs (11)	A	4 (36.3%)	< 60	7/10	A	2 (18.2%)	> 1 h/1d	7/10
	UA	2 (18.2%)	< 60	1/10 7/10	–	–	–	–
	U	2 (18.2%)	< 60	7/10	–	–	–	–
	ER	1 (9.1%)	< 60	7/10	–	–	–	–
Local anesthetics (1)	MPE	1 (100%)	10	2 ml	–	–	–	–

1/10–2/10–7/10: fractioned dose on first day of DPT

*TI* time interval

*m* minutes

*d* days of drugs administration

*h* hours from the last dose

*A* Angioedema, *ER* Erythematous Rash, *U* Urticaria, *MPE* Maculopapular exanthema, *UA* Urticaria+ Angioedema, *Ana* anaphylaxis, *ED* exfoliative dermatitis

^a^72 h after discontinuation (similar to a history of reactions)

During the DPTs with BLs, delayed reactions were more common than the immediate ones, in particular, the most frequently observed was represented by MPE in 25% cases. Four positive reactions, including one episode of anaphylaxis (urticaria plus hypotension requiring intramuscular administration of adrenaline), were observed despite all previous in vitro and in vivo tests being negative. Urticaria was the most common reaction during DPTs with macrolides (66.7%). With the NSAIDs, IRs occurred with a higher frequency, and angioedema (54.5%) was the most frequently observed. One patient had MPE after a DPT with mepivacaine.

Finally, we compared the reported clinical history of each patient with reactions occurring during the DPTs (Table 8). By analyzing the timing of the BL reactions, we obtained an overlapping of 20% (4/20) and 60% (12/20), for IRs and non-IRs respectively, and the symptoms in 45% (9/20) of patients were comparable; taken together, the percentage was 35% (7/20). Patients in the NSAIDs group had a percentage of 81% (9/11) for IR and 9.1% (1/11) for non-IR timing, and as regards the symptoms only 36.3% (4/11) had similar reactions, with very few patients, considering both clinical history and reaction, demonstrating an overlapping of 27.3% (3/11).

**Table 8:** Comparatives data of clinical history and reactions occurring during DPTs

	CLINICAL HISTORY			REACTION DURING DPTs		
Culprit	IRs	non-IRs	Reaction	IRs	non-IRs	Reaction
Amoxicillin- Clavulanic Acid	X		UA	X		U
		X	ER	X		U
	X		ER		X	ED
	X		U		X	U
		X	ER		X	ER
		X	MPE		X	ER
		X	UA		X	MPE
		X	U		X	MPE
		X	UA		X	U
		X	U		X	U
	X		U	X		Ana
		X	ER		X	ER
	X		U	X		U
	X		ER	X		ER
		X	MPE		X	MPE
		X	U	X		U
		X	MPE		X	ER+ Diarrhea
		X	MLR		X	ER
		X	ER		X	ER
		X	U		X	MPE
Clarithromycin	X		U + C	X		U
	X		U + C	X		ER + D
Azithromycin	X		U	X		U
Paracetamol	X		MPE	X		U
	X		ER	X		ER
Ibuprofen	X		UA	X		A
	X		UA	X		U
	X		U + C	X		UA
	X		U	X		A
	X		U	X		A
	X		A	X		A
	X		A		X	A
Local anesthetics		X	A		X	A
	X		U	X		UA

*ER* Erythematous Rash, *U* Urticaria, *MPE* Maculopapular exanthema, *A* Angioedema, *UA* Urticaria+ Angioedema, *Ana* anaphylaxis, *MLR* measles-like rash, *C* cough, *ED* exfoliative dermatitis

## Discussion

BLs are the most commonly prescribed antibiotics in children, and the majority of patients tested reported a history of reactions to this category of antibiotics. We had confirmation of hypersensitivity in 8% of examined cases, in line with data reported in literature (Caubet et al. observed a rate of 6.8% [4] and Zambonino et al. confirmed the diagnosis of BL hypersensitivity in 7.92% of cases [7]). Considering the reaction timing criteria, we had confirmation of hypersensitivity ranging between 8.1% (non- IRs) and 9.4% (IRs).

Non-IRs had positive results in 8.1% of patients, in line with data reported by Atanaskovic et al. (7.4%) [8] and Zambonino et al. (7.39%) [7]. A recent study by Lezmi et al. recorded 11.4% of hypersensitivity diagnoses for non-IRs [9].

Most of the patients were negative in the investigations; these data could be explained by the possible role of viruses as triggers of skin reactions, especially in cases of non-IRs. Many delayed reactions, in particular, urticaria and MPEs might be closely related to viruses [10]. In fact, as already reported in literature, viral infections are the most common causes of urticaria in childhood [11]. In this regard, it would be advisable to investigate a possible concomitant infection in the acute phase in order to exclude it wherever possible.

NSAIDs in our patients were the most common cause of anaphylaxis as reported in other studies [12–14], with ibuprofen the most frequently involved drug (46.6%). This percentage is in line with data referred previously by Blanca-Lopez et al. (41.2%) [15]. We had three cases with a clinical history of anaphylaxis to ketoprofen (3/15), in two cases the DPT with ASA was performed with negative results. Both were provoked with paracetamol and were negative, and in this group one patient was tested for ibuprofen with negative results. In the study by Blanca Lopez et al. [15] 38% of patients who had reactions to naproxen or dexketoprofen, tolerated ibuprofen. DPTs were not performed in 8 patients, but 7/8 patients tolerated paracetamol after the previous reaction to ibuprofen (6/7) or ketoprofen (1/7); in fact, paracetamol is a weak inhibitor of cyclooxygenase-1 (COX- 1) and it rarely causes symptoms in subjects with non-selective hypersensitivity. The low number of ASA DPTs performed was due to the fact that use of this drug is not recommended in children aged < 12 years. The majority of DPT reactions to NSAIDs were immediate and within 1 h after drug administration, especially in case of angioedema.

So far, very few studies have investigated reactions to vancomycin and quinolones at a pediatric age [16]. In our study a small number of patients was also tested for reactions to glycopeptides and quinolones.

Regarding the use of STs for testing antibiotic hypersensitivity, especially BLs, it is well known that they have diagnostic importance for IRs, but are burdened by a low sensitivity for non-IRs [4, 17]. Our study confirmed the low sensitivity of STs in non-IR cases (2.1%), with a relatively higher sensitivity of STs for IRs (4.6%). In the study of Caubet et al. 96 patients were evaluated with a higher rate of positive DPTs in those with positive immediate-reading IDTs; otherwise there were no positive delayed-reading IDTs in enrolled patients, including fourteen with positive DPTs. These data underline a low overall sensitivity and a higher specificity for immediate-reading IDTs [18]. In another study, Ponvert et al. confirmed the diagnosis of drug hypersensitivity using STs in 86% of IRs and 33.8% of non-IRs [17].

Our data emphasize the diagnostic value of STs for IRs. In fact, in case of anaphylaxis we had 4/11 positive ST results. Conversely, it must be noted that we had a positive DPT with an anaphylactic reaction an hour and a half after amoxicillin-clavulanic acid administration in a patient with all negative STs. Thus, despite negative ST results, by performing a DPT it was possible to make a correct diagnosis. At the same time, with the exclusion of SCARs, the performing of DPTs is mandatory for ensuring a confident diagnosis and a 5-day prolonged challenge for non-IRs is closely related to hypersensitivity confirmation (4.7% IRs vs. 6.2% non- IRs).

As regards macrolides, STs were positive in 21.3% of children. In a previous study we showed that 15.5% (9/58) of patients with reactions to clarithromycin and 47.3% (9/19) with previous reactions to azithromycin, had positive STs [19].

A confirmation of immediate hypersensitivity with DPT was obtained in 4.9% of patients with a history of IRs. A history of anaphylaxis induced by clarithromycin was recorded in 3 patients, 2/3 had negative STs and underwent DPT because of an unconvincing clinical history. Both reacted after the last fractioned dose (7/10), the first with urticaria, the second patient with gastrointestinal symptoms followed by an erythematous rash. In literature a few cases of anaphylaxis induced by macrolides have been described, Mori et al. analyzed three cases, two involving azithromycin, the third with azithromycin and clarithromycin [20]. Ben-Shoshan et al. reported one case related to clarithromycin in a child suffering from asthma during an episode of bronchopneumonia [21].

Overall, the diagnosis of hypersensitivity was confirmed in a higher number of cases in the macrolide group (30.7% in IRs and 22.8% in non-IRs) compared with the BL group (9.4% in IRs and 9.1% in non-IRs) but it must be taken in consideration that the number of patients investigated for macrolides was much lower than that of patients investigated for BLs.. When comparing these results with BL, we can hypothesize that the higher percentages observed in the macrolide group could be due to the lower number of patients investigated.

This study shows how the diagnosis of hypersensitivity was significantly higher when allergy testing was performed within 12 months after the onset of the reaction. This highlights the need for general pediatricians to send children to the allergy unit for a complete evaluation as soon as possible after a suspected drug reaction, since the possibility of having a confirmed diagnosis seems to be higher.

Severe reactions were more frequently associated with BLs. A study on a large group of SCAR cases reported penicillin followed by cephalosporins as the major culprits of SJS, SJS/TEN and acute generalized exanthematous pustulosis (AGEP); in this paper DRESS was more frequently linked with glycopeptides [22].

As in the study of Zambonino et al. [7], exanthema was the most common reaction after the DPTs, followed by urticaria; comparisons between the history of reactions and DPT reactions gave a percentage of 93.8% in a group of 783 patients. In our study the percentage of overlapping symptoms was lower (45%).

Immune-mediated reactions to local anesthetics are very rare, with an estimated percentage of less than 1% [23]. In our study only one patient was positive to the DPT. In a study based on a large cohort of patients (n:168), only including 8 children, no DPTs were positive [24].

Gomes et al. (2007) [25] tested 34 children with a history of drug allergy, conducting a complete allergy work-up. After in vivo, in vitro and drug provocation tests, a diagnosis of hypersensitivity was only confirmed in 3/34 (8.8%) of patients (one with a positive ST and two with positive DPTs with the culprit drug), which is the same percentage observed in our study. The classes of suspected drugs were similar to ours, with antibiotics (first of all BLs) and NSAIDs the most frequently reported. Non-IRs were more numerous than IRs, with cutaneous reactions the most frequently reported symptoms, exactly the same as in our series.

Erkocoglu et al. (2013) [26] performed a study on 101 children with suspected drug hypersensitivity reactions. In this group of patients, the suspected drugs were first antibiotics, followed by NSAIDs, with skin manifestations the most frequently reported symptoms. After a complete allergy investigation, only 9.4% of the suspected reactions were confirmed as real hypersensitivities by positive skin tests (3 in case of penicillins) or by positive DPTs (4 in case of NSAIDs and ferrous glycine sulfate), which is in line with percentage reported in our study.

Rubio et al. (2011) [27] observed a confirmed drug hypersensitivity in 10.6% of children tested with allergy tests. The classes of suspected drugs were antibiotics, first of all, BLs and NSAIDs.

Sousa Pinto et al. (2017) [28] recently carried out an extensive systematic review for assessing the prevalence of self-reported drug allergy in adults and children. Focusing on studies performed on the pediatric population, the rate of confirmed hypersensitivity after allergy work-up was reported to be 7.7%.

## Conclusion

Hypersensitivity to antibiotics in children is an important topic of debate, because overdiagnosis is quite common, especially in this age group. This could have an impact not only on public health, but also on antibiotic resistance, a condition already caused by over-administration of these drugs. It is important to raise awareness in primary care physicians for allowing to exclude hypersensitivity with an appropriate allergy work-up. It is interesting to note that in children this kind of reaction commonly involves infectious agents which must be ruled out where possible.

NSAIDs are frequently used drugs in children. In case of suspected hypersensitivity, it is essential to exclude this or otherwise ensure the possibility of using a safe alternative drug for pain or fever management. DPTs play a crucial role due to the variety of possible mechanisms involved in NSAIDs reactions.

Allergy to local anesthetics is not common, however, if there is a history of reactions, it is important to exclude other possible agents such as latex.

In general, a complete drug-allergy work-up is mandatory in order to obtain a confident diagnosis. For this reason, qualified personnel and settings are necessary in allergy units dealing with drug allergies in order to facilitate the treatment of severe reactions.

## Data Availability

The datasets during and/or analysed during the current study available from the corresponding author on reasonable request.

## Abbreviations

AGEP: Acute generalized exanthematous pustulosis
ASA: Acetylsalicylic acid
BLs: Beta lactams
DHRs: Drug Hypersensitivity Reactions
DPT: Drug provocation test
DRESS: Drug Reaction with Eosinophilia and Systemic Symptoms
IDT: Intradermal test
IR: An immediate reaction
MPEs: Maculopapular exanthemas
non-IR: non-immediate reaction
NSAIDs: Non-steroidal anti-inflammatory drugs
PT: Patch tests
SCARs: Severe cutaneous adverse reactions
SJS/TEN: Steven Johnson Syndrome/Toxic Epidermal Necrolysis
SPT: Skin prick test
